# *trans*-Chlorido­(4-fluoro­benzene­thiol­ato-κ*S*)bis­(tri­phenyl­phosphane-κ*P*)palladium(II) methanol hemisolvate

**DOI:** 10.1107/S1600536814002499

**Published:** 2014-02-12

**Authors:** Alcives Avila-Sorrosa, Ericka Santacruz-Juárez, Alicia Reyes-Arellano, Reyna Reyes-Martínez, David Morales-Morales

**Affiliations:** a Escuela Nacional de Ciencias Biológicas, IPN, Departamento de Química Orgánica, Caprio y Plan de Ayala S/N, Colonia Santo Tomás, 11340 México DF, Mexico; b Universidad Politécnica de Tlaxcala, Av. Universidad Politécnica de Tlaxcala No. 1, San Pedro Xalcaltzinco Municipio de Tepeyanco, Tlaxcala, CP 90180, Mexico; c Instituto de Química, Universidad Nacional Autónoma de México, Circuito exterior, Ciudad Universitaria, México, DF 04510, Mexico

## Abstract

The title compound, [Pd(SC_6_H_4_F-*p*)Cl(PPh_3_)_2_]·0.5CH_3_OH, features a Pd^II^ complex with two tri­phenyl­phosphane (PPh_3_) ligands arranged in a *trans* conformation, with one chloride and one 4-fluoro­benzene­thiol­ate ligand completing the coordination sphere, giving rise to a slightly distorted square-planar geometry of the Pd^II^ ion. The methanol solvent mol­ecule is disordered about an inversion centre with an occupancy of 0.25 for each molecule. In the crystal, weak C—H⋯Cl hydrogen-bonding inter­actions between the complex mol­ecules generate chain frameworks parallel to [010].

## Related literature   

For palladium complexes in catalysis, see: Frisch & Beller (2005 ▶); Yin & Liebscher (2007 ▶); Knochel & Singer (1993 ▶); Surry & Buchwald (2008 ▶). For related compounds, see: Jones *et al.* (2000 ▶); Alvarez-Larena *et al.* (1993 ▶).
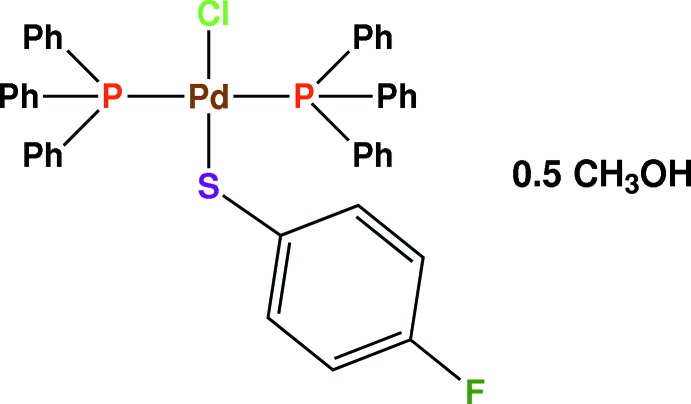


## Experimental   

### 

#### Crystal data  

[Pd(C_6_H_4_FS)Cl(C_18_H_15_P)_2_]·0.5CH_4_O
*M* *_r_* = 809.56
Monoclinic, 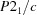

*a* = 18.9189 (16) Å
*b* = 9.6909 (8) Å
*c* = 21.6179 (18) Å
β = 106.212 (1)°
*V* = 3805.8 (6) Å^3^
*Z* = 4
Mo *K*α radiation
μ = 0.73 mm^−1^
*T* = 298 K
0.30 × 0.20 × 0.08 mm

#### Data collection  

Bruker SMART APEX CCD diffractometer
Absorption correction: multi-scan (*SADABS*; Sheldrick, 2008 ▶) *T*_min_ = 0.822, *T*_max_ = 0.947
30189 measured reflections
6950 independent reflections
5392 reflections with *I* > 2σ(*I*)
*R*_int_ = 0.052

#### Refinement  

*R*[*F* ^2^ > 2σ(*F* ^2^)] = 0.043
*wR*(*F* ^2^) = 0.110
*S* = 1.02
6950 reflections
469 parameters
33 restraints
H-atom parameters constrained
Δρ_max_ = 0.57 e Å^−3^
Δρ_min_ = −0.28 e Å^−3^

### 

Data collection: *SMART* (Bruker, 2007 ▶); cell refinement: *SAINT* (Bruker, 2007 ▶); data reduction: *SAINT*; program(s) used to solve structure: *SHELXTL* (Sheldrick, 2008 ▶); program(s) used to refine structure: *SHELXL97* (Sheldrick, 2008 ▶); molecular graphics: *ORTEP-3 for Windows* (Farrugia, 2012 ▶) and *DIAMOND* (Brandenburg, 2006 ▶); software used to prepare material for publication: *SHELXTL*) and *PLATON* (Spek, 2009 ▶).

## Supplementary Material

Crystal structure: contains datablock(s) I. DOI: 10.1107/S1600536814002499/hp2065sup1.cif

Structure factors: contains datablock(s) I. DOI: 10.1107/S1600536814002499/hp2065Isup2.hkl

CCDC reference:

Additional supporting information:  crystallographic information; 3D view; checkCIF report

## Figures and Tables

**Table 1:** Hydrogen-bond geometry (Å, °)

*D*—H⋯*A*	*D*—H	H⋯*A*	*D*⋯*A*	*D*—H⋯*A*
C34—H34⋯Cl1^i^	0.93	2.99	3.631 (5)	127
C35—H35⋯Cl1^i^	0.93	2.97	3.616 (4)	128

Symmetry code: (i) 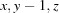.

**Fig. 1.**
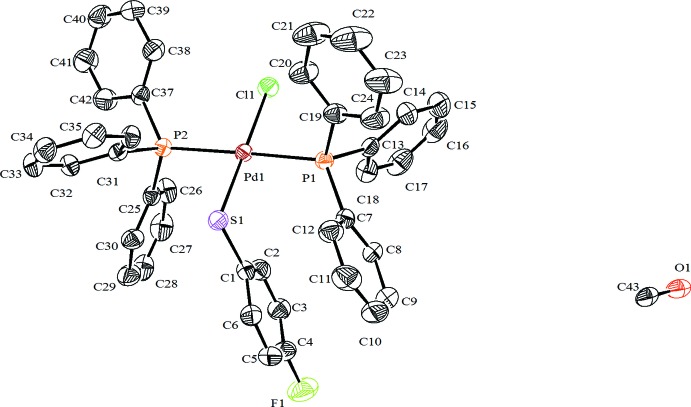
Molecular structure of the title compound showing the atom-labelling and displacement ellipsoids drawn at 40% probability level. The hydrogen atoms were omitted.

**Fig. 2.**
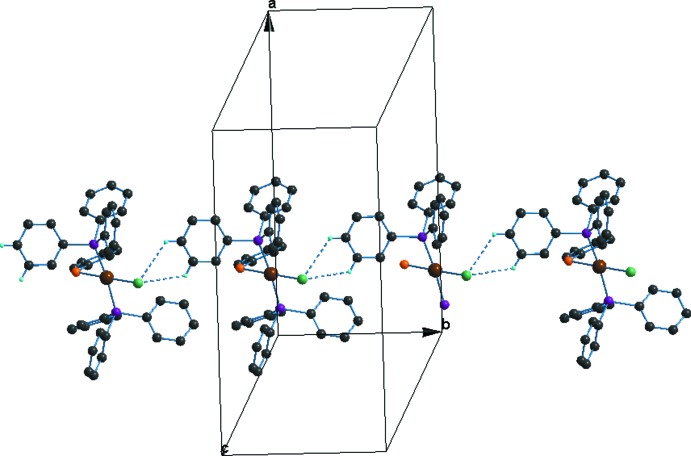
A portion of the crystal structure showing weak hydrogen bonds by dashed lines.

## supplementary crystallographic information

### 1. Introduction

The high efficiency of palladium complexes in C—C cross coupling reactions is well known, for example in the Suzuki-Miyaura (Frisch & Beller, 2005), Mizoroki-Heck (Yin & Liebscher, 2007) and Negishi reactions (Knochel & Singer, 1993), as well as for the formation of C-Heteroatom bonds as in the Buchwald–Hartwig couplings (Surry & Buchwald, 2008). In this context, different research groups have devoted their research efforts to the synthesis of new palladium complexes with a variety of ligands. Therefore, in this opportunity we would like to report the crystal structure of the Pd^II^ complex *trans*-[Pd(SC_6_H_4_F-*p*)(Cl)(PPh_3_)_2_] ·0.5CH_3_OH.

### 2.1. Synthesis and crystallization

To a suspension consisting of PdCl_2_ (0.072 g, 0.41 mmol) and Na_2_CO_3_ (0.050 g, 0.0471 mmol) in 15 mL of toluene was added dropwise under magnetic stirring a toluene solution (5 mL) of an isomeric mix of (3-ethenyl­benzyl)(4-fluoro­phenyl)­sulfane and (4-ethenyl­benzyl)(4-fluoro­phenyl)­sulfane (60:40) (0.100 g, 0.41 mmol). The resulting mixture was set to reflux for 4 hours. After this time the reaction mixture was allowed to cool down to room temperature, filtered and the solid product washed with CHCl_3_. The insoluble reddish solid was then suspended in 15 mL of CHCl_3_ and then treated with Ph_3_P (0.215 g, 820 mmol). The reaction was allowed to proceed under stirring until the solid was completely dissolved. This solution was then filtered through a short plug of celite and CH_3_OH was added to promote precipitation. Slow evaporation of the solvent at room temperature produced brown crystals for X-ray diffraction analysis of *trans*-[Pd(SC_6_H_4_F-*p*)(Cl)(PPh_3_)_2_] ·0.5CH_3_OH.

### 2.2. Refinement

Crystal data, data collection and structure refinement details are summarized in Table 2. H atoms were included in calculated positions (C—H = 0.93 Å for aromatic H) and refined using a riding model with U_iso_(H) = 1.2 U_eq_ of carrier atoms. The CH_3_OH solvent is disordered over two sets of sites in a 0.25 : 0.25 ratio.

### 3. Results and discussion

The compound *trans*-[Pd(SC_6_H_4_F-*p*)(Cl)(PPh_3_)_2_] ·0.5CH_3_OH crystallized in a monoclinic system including the complex *trans*-[Pd(SC_6_H_4_F-*p*)(Cl)(PPh_3_)_2_] and half molecule of methanol in the asymmetric unit. The Pd^2+^ ion exhibits a slightly distorted square-planar PdClSP_2_ geometry, with one chloride and one *p*-fluoro­benzene­thiol­ate ligand and two molecules of tri­phenyl­phosphane in *trans* conformation completing the coordination sphere. The bond distances Pd—Cl and Pd—S are 2.3269 (11) and 2.2977 (11) Å, respectively, and the Pd—P distances are 2.3432 (11) and 2.3342 (11) Å. These distances are comparable to those found in the structures of the related compounds *trans*-[Pd(SC_6_H_4_Cl-*p*)(Cl)(PPh_3_)_2_] (Jones *et al.*, 2000) and trans-[Pd(SC_6_H_5_)(Cl)(PPh_3_)_2_] (Alvarez-Larena *et al.*, 1993).

The *p*-fluoro­benzene­thiol­ate presents an intra­molecular π-π inter­action with one phenyl ring of the tripehylphosphane ligand. The distance between centroids *Cg(C1—C6)*-*Cg(C7—C12)* is 3.677 (3) Å. The structure is stabilized by weak C—H···Cl hydrogen bonding inter­actions between the complex molecules generate chain frameworks parallel to [010]. The methanol molecule is disordered over two positions in a 0.25:0.25 ratio.

### Crystal data

**Table:**

[Pd(C_6_H_4_FS)Cl(C_18_H_15_P)_2_]·0.5CH_4_O	*F*(000) = 1652
*M**_r_* = 809.56	*D*_x_ = 1.413 Mg m^−^^3^
Monoclinic, *P*2_1_/*c*	Mo *K*α radiation, λ = 0.71073 Å
*a* = 18.9189 (16) Å	Cell parameters from 8442 reflections
*b* = 9.6909 (8) Å	θ = 2.3–25.1°
*c* = 21.6179 (18) Å	µ = 0.73 mm^−^^1^
β = 106.212 (1)°	*T* = 298 K
*V* = 3805.8 (6) Å^3^	Plates, dark-pink
*Z* = 4	0.30 × 0.20 × 0.08 mm

### Data collection

**Table:**

Bruker SMART APEX CCD diffractometer	6950 independent reflections
Radiation source: fine-focus sealed tube	5392 reflections with *I* > 2σ(*I*)
Graphite monochromator	*R*_int_ = 0.052
Detector resolution: 8.333 pixels mm^-1^	θ_max_ = 25.4°, θ_min_ = 2.0°
ω–scans	*h* = −22→22
Absorption correction: multi-scan (*SADABS*; Sheldrick, 2008)	*k* = −11→11
*T*_min_ = 0.822, *T*_max_ = 0.947	*l* = −26→26
30189 measured reflections	

### Refinement

**Table:**

Refinement on *F*^2^	Primary atom site location: structure-invariant direct methods
Least-squares matrix: full	Secondary atom site location: difference Fourier map
*R*[*F*^2^ > 2σ(*F*^2^)] = 0.043	Hydrogen site location: mixed
*wR*(*F*^2^) = 0.110	H-atom parameters constrained
*S* = 1.02	*w* = 1/[σ^2^(*F*_o_^2^) + (0.0613*P*)^2^] where *P* = (*F*_o_^2^ + 2*F*_c_^2^)/3
6950 reflections	(Δ/σ)_max_ < 0.001
469 parameters	Δρ_max_ = 0.57 e Å^−^^3^
33 restraints	Δρ_min_ = −0.28 e Å^−^^3^

### Special details

**Table:**

Geometry. All e.s.d.'s (except the e.s.d. in the dihedral angle between two l.s. planes) are estimated using the full covariance matrix. The cell e.s.d.'s are taken into account individually in the estimation of e.s.d.'s in distances, angles and torsion angles; correlations between e.s.d.'s in cell parameters are only used when they are defined by crystal symmetry. An approximate (isotropic) treatment of cell e.s.d.'s is used for estimating e.s.d.'s involving l.s. planes.

### Fractional atomic coordinates and isotropic or equivalent isotropic displacement parameters (Å^2^)

**Table:**

	*x*	*y*	*z*	*U*_iso_*/*U*_eq_	Occ. (<1)
Pd1	0.24393 (2)	0.02208 (3)	0.14849 (2)	0.04003 (11)	
Cl1	0.19542 (6)	0.18164 (10)	0.06730 (5)	0.0558 (3)	
S1	0.29295 (6)	−0.13516 (10)	0.22852 (5)	0.0519 (2)	
P1	0.15593 (5)	0.09096 (10)	0.19997 (5)	0.0437 (2)	
P2	0.33009 (5)	−0.06767 (10)	0.10028 (5)	0.0424 (2)	
F1	0.44844 (17)	0.1686 (4)	0.46211 (14)	0.1094 (11)	
C1	0.3380 (2)	−0.0375 (4)	0.29652 (19)	0.0517 (10)	
C2	0.3650 (2)	0.0954 (5)	0.2943 (2)	0.0615 (11)	
H2	0.3584	0.1383	0.2547	0.074*	
C3	0.4012 (2)	0.1645 (5)	0.3494 (2)	0.0658 (12)	
H3	0.4181	0.2540	0.3473	0.079*	
C4	0.4120 (2)	0.1002 (6)	0.4074 (2)	0.0736 (13)	
C5	0.3885 (3)	−0.0311 (6)	0.4122 (2)	0.0740 (13)	
H5	0.3977	−0.0742	0.4521	0.089*	
C6	0.3510 (2)	−0.0987 (5)	0.3569 (2)	0.0623 (11)	
H6	0.3338	−0.1876	0.3599	0.075*	
C7	0.1691 (2)	0.0487 (4)	0.28479 (19)	0.0495 (9)	
C8	0.1943 (2)	0.1436 (5)	0.33324 (19)	0.0658 (12)	
H8	0.2035	0.2338	0.3230	0.079*	
C9	0.2059 (3)	0.1061 (7)	0.3973 (2)	0.0877 (16)	
H9	0.2233	0.1714	0.4295	0.105*	
C10	0.1924 (3)	−0.0239 (8)	0.4135 (3)	0.098 (2)	
H10	0.2003	−0.0483	0.4565	0.117*	
C11	0.1666 (3)	−0.1204 (6)	0.3652 (3)	0.0883 (16)	
H11	0.1560	−0.2095	0.3759	0.106*	
C12	0.1564 (2)	−0.0862 (5)	0.3015 (2)	0.0677 (12)	
H12	0.1411	−0.1529	0.2696	0.081*	
C13	0.1360 (2)	0.2754 (4)	0.19518 (17)	0.0507 (9)	
C14	0.0647 (3)	0.3255 (5)	0.1726 (2)	0.0717 (13)	
H14	0.0255	0.2641	0.1600	0.086*	
C15	0.0516 (4)	0.4662 (6)	0.1687 (3)	0.1000 (19)	
H15	0.0037	0.4986	0.1526	0.120*	
C16	0.1081 (5)	0.5574 (6)	0.1883 (3)	0.099 (2)	
H16	0.0987	0.6518	0.1858	0.119*	
C17	0.1799 (4)	0.5098 (5)	0.2121 (3)	0.0893 (17)	
H17	0.2186	0.5716	0.2266	0.107*	
C18	0.1931 (3)	0.3675 (5)	0.2140 (2)	0.0670 (12)	
H18	0.2412	0.3350	0.2281	0.080*	
C19	0.0685 (2)	0.0080 (4)	0.1601 (2)	0.0574 (11)	
C20	0.0565 (3)	−0.0437 (5)	0.0987 (2)	0.0761 (14)	
H20	0.0931	−0.0360	0.0779	0.091*	
C21	−0.0097 (3)	−0.1070 (7)	0.0675 (3)	0.103 (2)	
H21	−0.0182	−0.1391	0.0255	0.123*	
C22	−0.0624 (3)	−0.1214 (8)	0.0995 (3)	0.117 (2)	
H22	−0.1064	−0.1657	0.0793	0.141*	
C23	−0.0514 (3)	−0.0726 (8)	0.1596 (3)	0.116 (2)	
H23	−0.0876	−0.0830	0.1808	0.139*	
C24	0.0138 (3)	−0.0069 (6)	0.1899 (3)	0.0874 (17)	
H24	0.0208	0.0278	0.2313	0.105*	
C25	0.4207 (2)	−0.0086 (4)	0.14525 (19)	0.0488 (9)	
C26	0.4425 (2)	0.1222 (4)	0.1350 (2)	0.0678 (12)	
H26	0.4125	0.1769	0.1029	0.081*	
C27	0.5096 (3)	0.1733 (5)	0.1729 (3)	0.0836 (15)	
H27	0.5242	0.2621	0.1657	0.100*	
C28	0.5541 (3)	0.0943 (6)	0.2205 (3)	0.0838 (16)	
H28	0.5993	0.1287	0.2447	0.101*	
C29	0.5330 (3)	−0.0320 (6)	0.2322 (2)	0.0754 (14)	
H29	0.5628	−0.0841	0.2655	0.091*	
C30	0.4664 (2)	−0.0865 (5)	0.1947 (2)	0.0625 (11)	
H30	0.4525	−0.1752	0.2028	0.075*	
C31	0.33212 (19)	−0.2558 (4)	0.09877 (16)	0.0434 (8)	
C32	0.3926 (2)	−0.3279 (4)	0.09171 (19)	0.0536 (10)	
H32	0.4351	−0.2801	0.0910	0.064*	
C33	0.3907 (3)	−0.4696 (4)	0.0857 (2)	0.0641 (11)	
H33	0.4318	−0.5175	0.0817	0.077*	
C34	0.3277 (3)	−0.5392 (4)	0.0857 (2)	0.0674 (12)	
H34	0.3263	−0.6347	0.0815	0.081*	
C35	0.2672 (3)	−0.4707 (4)	0.0917 (2)	0.0601 (11)	
H35	0.2246	−0.5193	0.0913	0.072*	
C36	0.2691 (2)	−0.3287 (4)	0.09834 (18)	0.0530 (10)	
H36	0.2278	−0.2819	0.1025	0.064*	
C37	0.3254 (2)	−0.0314 (4)	0.01670 (19)	0.0487 (9)	
C38	0.2591 (3)	−0.0288 (4)	−0.0293 (2)	0.0655 (12)	
H38	0.2160	−0.0342	−0.0167	0.079*	
C39	0.2546 (3)	−0.0181 (5)	−0.0945 (2)	0.0776 (14)	
H39	0.2090	−0.0179	−0.1252	0.093*	
C40	0.3169 (3)	−0.0081 (5)	−0.1131 (3)	0.0799 (15)	
H40	0.3138	0.0013	−0.1566	0.096*	
C41	0.3848 (3)	−0.0118 (6)	−0.0684 (3)	0.0891 (17)	
H41	0.4274	−0.0064	−0.0817	0.107*	
C42	0.3896 (3)	−0.0232 (5)	−0.0041 (2)	0.0780 (14)	
H42	0.4356	−0.0257	0.0260	0.094*	
O1	0.0542 (7)	1.0384 (15)	0.5041 (6)	0.077 (4)	0.25
H1	0.0112	1.0626	0.4896	0.115*	0.25
C43	0.0682 (13)	0.889 (3)	0.5018 (13)	0.071 (4)	0.25
H43A	0.0231	0.8397	0.4971	0.086*	0.25
H43B	0.1036	0.8617	0.5409	0.086*	0.25
H43C	0.0869	0.8698	0.4658	0.086*	0.25
O1B	0.0386 (8)	0.928 (2)	0.5026 (8)	0.084 (4)	0.25
H1B	0.0521	0.9888	0.4820	0.125*	0.25
C43B	0.0690 (11)	0.808 (2)	0.4885 (10)	0.079 (4)	0.25
H43D	0.0340	0.7605	0.4542	0.094*	0.25
H43E	0.0821	0.7499	0.5260	0.094*	0.25
H43F	0.1123	0.8284	0.4752	0.094*	0.25

### Atomic displacement parameters (Å^2^)

**Table:**

	*U*^11^	*U*^22^	*U*^33^	*U*^12^	*U*^13^	*U*^23^
Pd1	0.04087 (17)	0.03949 (17)	0.03993 (17)	−0.00118 (12)	0.01160 (12)	−0.00155 (12)
Cl1	0.0686 (6)	0.0477 (6)	0.0503 (6)	0.0034 (5)	0.0151 (5)	0.0052 (4)
S1	0.0627 (6)	0.0458 (5)	0.0468 (6)	0.0065 (5)	0.0147 (5)	0.0020 (4)
P1	0.0414 (5)	0.0484 (6)	0.0416 (5)	−0.0008 (4)	0.0119 (4)	−0.0012 (4)
P2	0.0429 (5)	0.0412 (5)	0.0446 (5)	−0.0017 (4)	0.0147 (4)	−0.0026 (4)
F1	0.100 (2)	0.147 (3)	0.0691 (19)	−0.022 (2)	0.0042 (17)	−0.0266 (19)
C1	0.047 (2)	0.056 (2)	0.053 (2)	0.0114 (18)	0.0148 (18)	0.0026 (19)
C2	0.055 (2)	0.069 (3)	0.060 (3)	0.003 (2)	0.016 (2)	0.006 (2)
C3	0.054 (3)	0.076 (3)	0.064 (3)	−0.011 (2)	0.011 (2)	−0.008 (2)
C4	0.058 (3)	0.102 (4)	0.053 (3)	−0.002 (3)	0.002 (2)	−0.016 (3)
C5	0.072 (3)	0.095 (4)	0.049 (3)	0.017 (3)	0.008 (2)	0.007 (3)
C6	0.069 (3)	0.066 (3)	0.052 (3)	0.011 (2)	0.018 (2)	0.005 (2)
C7	0.046 (2)	0.059 (3)	0.046 (2)	0.0070 (18)	0.0166 (18)	0.0041 (19)
C8	0.074 (3)	0.078 (3)	0.045 (2)	0.021 (2)	0.015 (2)	−0.005 (2)
C9	0.089 (4)	0.118 (5)	0.054 (3)	0.032 (3)	0.015 (3)	−0.008 (3)
C10	0.099 (4)	0.146 (6)	0.051 (3)	0.033 (4)	0.027 (3)	0.027 (4)
C11	0.090 (4)	0.102 (4)	0.077 (4)	0.008 (3)	0.029 (3)	0.038 (3)
C12	0.068 (3)	0.077 (3)	0.057 (3)	0.000 (2)	0.017 (2)	0.007 (2)
C13	0.064 (3)	0.052 (2)	0.041 (2)	0.006 (2)	0.0214 (19)	−0.0009 (18)
C14	0.075 (3)	0.068 (3)	0.070 (3)	0.021 (2)	0.016 (2)	0.001 (2)
C15	0.123 (5)	0.085 (4)	0.088 (4)	0.049 (4)	0.024 (4)	0.004 (3)
C16	0.171 (7)	0.058 (3)	0.076 (4)	0.031 (4)	0.045 (4)	0.009 (3)
C17	0.134 (5)	0.067 (4)	0.076 (4)	−0.018 (3)	0.045 (4)	−0.008 (3)
C18	0.078 (3)	0.055 (3)	0.073 (3)	−0.003 (2)	0.029 (3)	0.000 (2)
C19	0.048 (2)	0.069 (3)	0.052 (3)	−0.0102 (19)	0.010 (2)	0.003 (2)
C20	0.067 (3)	0.094 (4)	0.064 (3)	−0.030 (3)	0.013 (2)	−0.005 (3)
C21	0.093 (4)	0.137 (5)	0.068 (3)	−0.055 (4)	0.005 (3)	−0.010 (3)
C22	0.084 (4)	0.173 (7)	0.083 (4)	−0.069 (4)	0.002 (3)	0.009 (4)
C23	0.054 (3)	0.208 (7)	0.083 (4)	−0.041 (4)	0.013 (3)	0.023 (5)
C24	0.053 (3)	0.145 (5)	0.063 (3)	−0.022 (3)	0.014 (2)	−0.003 (3)
C25	0.050 (2)	0.044 (2)	0.054 (2)	−0.0033 (17)	0.0162 (19)	−0.0112 (18)
C26	0.060 (3)	0.052 (3)	0.095 (3)	−0.006 (2)	0.027 (2)	−0.005 (2)
C27	0.078 (3)	0.062 (3)	0.118 (5)	−0.028 (3)	0.039 (3)	−0.026 (3)
C28	0.052 (3)	0.093 (4)	0.104 (4)	−0.022 (3)	0.019 (3)	−0.046 (4)
C29	0.060 (3)	0.095 (4)	0.066 (3)	−0.010 (3)	0.008 (2)	−0.022 (3)
C30	0.057 (3)	0.069 (3)	0.057 (3)	−0.011 (2)	0.009 (2)	−0.009 (2)
C31	0.048 (2)	0.042 (2)	0.039 (2)	−0.0035 (16)	0.0102 (16)	−0.0041 (16)
C32	0.047 (2)	0.053 (2)	0.061 (3)	0.0034 (18)	0.0162 (19)	0.001 (2)
C33	0.068 (3)	0.053 (3)	0.072 (3)	0.016 (2)	0.020 (2)	0.000 (2)
C34	0.089 (4)	0.042 (2)	0.071 (3)	0.001 (2)	0.023 (3)	−0.002 (2)
C35	0.070 (3)	0.046 (2)	0.067 (3)	−0.018 (2)	0.025 (2)	−0.006 (2)
C36	0.051 (2)	0.053 (2)	0.060 (3)	−0.0027 (18)	0.022 (2)	−0.0062 (19)
C37	0.057 (2)	0.042 (2)	0.049 (2)	0.0004 (18)	0.019 (2)	0.0006 (17)
C38	0.067 (3)	0.070 (3)	0.061 (3)	−0.004 (2)	0.019 (2)	−0.007 (2)
C39	0.081 (3)	0.093 (4)	0.053 (3)	0.010 (3)	0.008 (3)	−0.001 (2)
C40	0.109 (4)	0.080 (4)	0.056 (3)	0.009 (3)	0.031 (3)	0.007 (2)
C41	0.086 (4)	0.126 (5)	0.067 (3)	0.007 (3)	0.040 (3)	0.013 (3)
C42	0.061 (3)	0.112 (4)	0.064 (3)	0.004 (3)	0.021 (2)	0.007 (3)
O1	0.051 (6)	0.131 (10)	0.048 (5)	−0.026 (7)	0.014 (5)	0.006 (7)
C43	0.040 (7)	0.134 (10)	0.045 (6)	−0.025 (9)	0.020 (7)	0.000 (8)
O1B	0.055 (7)	0.153 (11)	0.048 (5)	−0.042 (8)	0.023 (6)	0.003 (7)
C43B	0.051 (7)	0.141 (12)	0.045 (8)	−0.036 (9)	0.016 (6)	0.022 (9)

### Geometric parameters (Å, º)

**Table:**

Pd1—S1	2.2976 (10)	C21—H21	0.9300
Pd1—Cl1	2.3269 (10)	C22—C23	1.345 (8)
Pd1—P2	2.3342 (10)	C22—H22	0.9300
Pd1—P1	2.3432 (10)	C23—C24	1.380 (7)
S1—C1	1.756 (4)	C23—H23	0.9300
P1—C13	1.824 (4)	C24—H24	0.9300
P1—C19	1.825 (4)	C25—C26	1.369 (5)
P1—C7	1.827 (4)	C25—C30	1.394 (6)
P2—C25	1.812 (4)	C26—C27	1.396 (6)
P2—C37	1.818 (4)	C26—H26	0.9300
P2—C31	1.824 (4)	C27—C28	1.368 (7)
F1—C4	1.364 (5)	C27—H27	0.9300
C1—C2	1.391 (6)	C28—C29	1.333 (7)
C1—C6	1.392 (6)	C28—H28	0.9300
C2—C3	1.371 (6)	C29—C30	1.398 (6)
C2—H2	0.9300	C29—H29	0.9300
C3—C4	1.362 (6)	C30—H30	0.9300
C3—H3	0.9300	C31—C36	1.384 (5)
C4—C5	1.362 (7)	C31—C32	1.385 (5)
C5—C6	1.374 (6)	C32—C33	1.380 (5)
C5—H5	0.9300	C32—H32	0.9300
C6—H6	0.9300	C33—C34	1.368 (6)
C7—C8	1.375 (6)	C33—H33	0.9300
C7—C12	1.394 (6)	C34—C35	1.362 (6)
C8—C9	1.389 (6)	C34—H34	0.9300
C8—H8	0.9300	C35—C36	1.384 (5)
C9—C10	1.351 (8)	C35—H35	0.9300
C9—H9	0.9300	C36—H36	0.9300
C10—C11	1.386 (8)	C37—C38	1.366 (6)
C10—H10	0.9300	C37—C42	1.410 (6)
C11—C12	1.376 (6)	C38—C39	1.394 (6)
C11—H11	0.9300	C38—H38	0.9300
C12—H12	0.9300	C39—C40	1.349 (7)
C13—C18	1.372 (6)	C39—H39	0.9300
C13—C14	1.388 (6)	C40—C41	1.376 (7)
C14—C15	1.384 (7)	C40—H40	0.9300
C14—H14	0.9300	C41—C42	1.371 (7)
C15—C16	1.361 (9)	C41—H41	0.9300
C15—H15	0.9300	C42—H42	0.9300
C16—C17	1.390 (9)	O1—C43	1.47 (2)
C16—H16	0.9300	O1—H1	0.8200
C17—C18	1.400 (7)	C43—H43A	0.9600
C17—H17	0.9300	C43—H43B	0.9600
C18—H18	0.9300	C43—H43C	0.9600
C19—C24	1.370 (6)	O1B—C43B	1.37 (2)
C19—C20	1.376 (6)	O1B—H1B	0.8199
C20—C21	1.389 (6)	C43B—H43D	0.9600
C20—H20	0.9300	C43B—H43E	0.9600
C21—C22	1.368 (8)	C43B—H43F	0.9600
			
S1—Pd1—Cl1	179.44 (4)	C22—C21—H21	120.5
S1—Pd1—P2	84.47 (4)	C20—C21—H21	120.5
Cl1—Pd1—P2	95.07 (4)	C23—C22—C21	121.0 (5)
S1—Pd1—P1	91.28 (4)	C23—C22—H22	119.5
Cl1—Pd1—P1	89.20 (4)	C21—C22—H22	119.5
P2—Pd1—P1	174.61 (4)	C22—C23—C24	119.8 (5)
C1—S1—Pd1	105.81 (14)	C22—C23—H23	120.1
C13—P1—C19	105.03 (19)	C24—C23—H23	120.1
C13—P1—C7	104.19 (17)	C19—C24—C23	121.2 (5)
C19—P1—C7	103.12 (19)	C19—C24—H24	119.4
C13—P1—Pd1	114.53 (12)	C23—C24—H24	119.4
C19—P1—Pd1	108.71 (14)	C26—C25—C30	118.4 (4)
C7—P1—Pd1	119.79 (12)	C26—C25—P2	119.2 (3)
C25—P2—C37	104.38 (18)	C30—C25—P2	122.1 (3)
C25—P2—C31	107.68 (17)	C25—C26—C27	119.9 (5)
C37—P2—C31	99.83 (16)	C25—C26—H26	120.1
C25—P2—Pd1	108.49 (12)	C27—C26—H26	120.1
C37—P2—Pd1	121.69 (13)	C28—C27—C26	120.7 (5)
C31—P2—Pd1	113.66 (12)	C28—C27—H27	119.6
C2—C1—C6	117.1 (4)	C26—C27—H27	119.6
C2—C1—S1	124.6 (3)	C29—C28—C27	120.1 (4)
C6—C1—S1	118.2 (3)	C29—C28—H28	119.9
C3—C2—C1	121.4 (4)	C27—C28—H28	119.9
C3—C2—H2	119.3	C28—C29—C30	120.4 (5)
C1—C2—H2	119.3	C28—C29—H29	119.8
C4—C3—C2	119.1 (4)	C30—C29—H29	119.8
C4—C3—H3	120.5	C25—C30—C29	120.4 (4)
C2—C3—H3	120.5	C25—C30—H30	119.8
C5—C4—C3	122.0 (4)	C29—C30—H30	119.8
C5—C4—F1	119.0 (5)	C36—C31—C32	118.6 (3)
C3—C4—F1	119.0 (5)	C36—C31—P2	119.2 (3)
C4—C5—C6	118.6 (4)	C32—C31—P2	121.9 (3)
C4—C5—H5	120.7	C33—C32—C31	120.7 (4)
C6—C5—H5	120.7	C33—C32—H32	119.6
C5—C6—C1	121.7 (4)	C31—C32—H32	119.6
C5—C6—H6	119.1	C34—C33—C32	119.4 (4)
C1—C6—H6	119.1	C34—C33—H33	120.3
C8—C7—C12	118.5 (4)	C32—C33—H33	120.3
C8—C7—P1	122.4 (3)	C35—C34—C33	121.0 (4)
C12—C7—P1	119.0 (3)	C35—C34—H34	119.5
C7—C8—C9	120.6 (5)	C33—C34—H34	119.5
C7—C8—H8	119.7	C34—C35—C36	119.8 (4)
C9—C8—H8	119.7	C34—C35—H35	120.1
C10—C9—C8	120.9 (5)	C36—C35—H35	120.1
C10—C9—H9	119.6	C35—C36—C31	120.4 (4)
C8—C9—H9	119.6	C35—C36—H36	119.8
C9—C10—C11	119.1 (5)	C31—C36—H36	119.8
C9—C10—H10	120.4	C38—C37—C42	117.7 (4)
C11—C10—H10	120.4	C38—C37—P2	120.4 (3)
C12—C11—C10	120.8 (5)	C42—C37—P2	121.3 (3)
C12—C11—H11	119.6	C37—C38—C39	121.4 (4)
C10—C11—H11	119.6	C37—C38—H38	119.3
C11—C12—C7	119.9 (5)	C39—C38—H38	119.3
C11—C12—H12	120.0	C40—C39—C38	119.7 (5)
C7—C12—H12	120.0	C40—C39—H39	120.1
C18—C13—C14	119.0 (4)	C38—C39—H39	120.1
C18—C13—P1	119.2 (3)	C39—C40—C41	120.7 (5)
C14—C13—P1	121.8 (3)	C39—C40—H40	119.7
C15—C14—C13	120.4 (5)	C41—C40—H40	119.7
C15—C14—H14	119.8	C42—C41—C40	119.9 (5)
C13—C14—H14	119.8	C42—C41—H41	120.0
C16—C15—C14	120.6 (6)	C40—C41—H41	120.0
C16—C15—H15	119.7	C41—C42—C37	120.5 (5)
C14—C15—H15	119.7	C41—C42—H42	119.7
C15—C16—C17	120.1 (5)	C37—C42—H42	119.7
C15—C16—H16	120.0	C43—O1—H1	115.9
C17—C16—H16	120.0	O1—C43—H43A	109.5
C16—C17—C18	119.0 (6)	O1—C43—H43B	109.5
C16—C17—H17	120.5	H43A—C43—H43B	109.5
C18—C17—H17	120.5	O1—C43—H43C	109.5
C13—C18—C17	120.9 (5)	H43A—C43—H43C	109.5
C13—C18—H18	119.6	H43B—C43—H43C	109.5
C17—C18—H18	119.6	C43B—O1B—H1B	106.1
C24—C19—C20	118.1 (4)	O1B—C43B—H43D	109.5
C24—C19—P1	121.9 (3)	O1B—C43B—H43E	109.5
C20—C19—P1	120.0 (3)	H43D—C43B—H43E	109.5
C19—C20—C21	120.9 (5)	O1B—C43B—H43F	109.5
C19—C20—H20	119.6	H43D—C43B—H43F	109.5
C21—C20—H20	119.6	H43E—C43B—H43F	109.5
C22—C21—C20	119.0 (5)		
			
Pd1—S1—C1—C2	−24.8 (4)	C19—C20—C21—C22	−2.0 (9)
Pd1—S1—C1—C6	158.4 (3)	C20—C21—C22—C23	1.5 (11)
C6—C1—C2—C3	−1.6 (6)	C21—C22—C23—C24	−0.1 (12)
S1—C1—C2—C3	−178.5 (3)	C20—C19—C24—C23	0.4 (8)
C1—C2—C3—C4	1.3 (6)	P1—C19—C24—C23	−178.6 (5)
C2—C3—C4—C5	0.6 (7)	C22—C23—C24—C19	−0.9 (10)
C2—C3—C4—F1	179.5 (4)	C37—P2—C25—C26	51.5 (4)
C3—C4—C5—C6	−1.9 (7)	C31—P2—C25—C26	156.9 (3)
F1—C4—C5—C6	179.2 (4)	Pd1—P2—C25—C26	−79.6 (3)
C4—C5—C6—C1	1.5 (7)	C37—P2—C25—C30	−135.2 (3)
C2—C1—C6—C5	0.3 (6)	C31—P2—C25—C30	−29.8 (4)
S1—C1—C6—C5	177.4 (3)	Pd1—P2—C25—C30	93.7 (3)
C13—P1—C7—C8	−28.2 (4)	C30—C25—C26—C27	1.2 (6)
C19—P1—C7—C8	−137.7 (3)	P2—C25—C26—C27	174.8 (3)
Pd1—P1—C7—C8	101.5 (3)	C25—C26—C27—C28	−0.2 (7)
C13—P1—C7—C12	154.7 (3)	C26—C27—C28—C29	−1.5 (8)
C19—P1—C7—C12	45.2 (4)	C27—C28—C29—C30	2.1 (8)
Pd1—P1—C7—C12	−75.6 (3)	C26—C25—C30—C29	−0.7 (6)
C12—C7—C8—C9	−0.7 (6)	P2—C25—C30—C29	−174.0 (3)
P1—C7—C8—C9	−177.8 (3)	C28—C29—C30—C25	−1.0 (7)
C7—C8—C9—C10	−0.5 (7)	C25—P2—C31—C36	147.5 (3)
C8—C9—C10—C11	0.0 (8)	C37—P2—C31—C36	−103.8 (3)
C9—C10—C11—C12	1.6 (8)	Pd1—P2—C31—C36	27.3 (3)
C10—C11—C12—C7	−2.8 (7)	C25—P2—C31—C32	−38.9 (4)
C8—C7—C12—C11	2.3 (6)	C37—P2—C31—C32	69.7 (3)
P1—C7—C12—C11	179.5 (4)	Pd1—P2—C31—C32	−159.1 (3)
C19—P1—C13—C18	−171.9 (3)	C36—C31—C32—C33	−1.3 (6)
C7—P1—C13—C18	80.0 (3)	P2—C31—C32—C33	−174.9 (3)
Pd1—P1—C13—C18	−52.7 (3)	C31—C32—C33—C34	1.1 (7)
C19—P1—C13—C14	7.6 (4)	C32—C33—C34—C35	−0.3 (7)
C7—P1—C13—C14	−100.4 (3)	C33—C34—C35—C36	−0.4 (7)
Pd1—P1—C13—C14	126.8 (3)	C34—C35—C36—C31	0.1 (6)
C18—C13—C14—C15	0.4 (7)	C32—C31—C36—C35	0.7 (6)
P1—C13—C14—C15	−179.1 (4)	P2—C31—C36—C35	174.5 (3)
C13—C14—C15—C16	−1.5 (8)	C25—P2—C37—C38	−162.9 (3)
C14—C15—C16—C17	0.4 (9)	C31—P2—C37—C38	85.9 (4)
C15—C16—C17—C18	1.6 (8)	Pd1—P2—C37—C38	−40.0 (4)
C14—C13—C18—C17	1.6 (6)	C25—P2—C37—C42	25.5 (4)
P1—C13—C18—C17	−178.8 (3)	C31—P2—C37—C42	−85.7 (4)
C16—C17—C18—C13	−2.7 (7)	Pd1—P2—C37—C42	148.4 (3)
C13—P1—C19—C24	−77.5 (4)	C42—C37—C38—C39	−0.1 (6)
C7—P1—C19—C24	31.4 (4)	P2—C37—C38—C39	−172.0 (4)
Pd1—P1—C19—C24	159.5 (4)	C37—C38—C39—C40	−1.0 (7)
C13—P1—C19—C20	103.6 (4)	C38—C39—C40—C41	1.6 (8)
C7—P1—C19—C20	−147.6 (4)	C39—C40—C41—C42	−1.1 (8)
Pd1—P1—C19—C20	−19.4 (4)	C40—C41—C42—C37	0.0 (8)
C24—C19—C20—C21	1.1 (8)	C38—C37—C42—C41	0.6 (7)
P1—C19—C20—C21	−179.9 (4)	P2—C37—C42—C41	172.4 (4)

### Hydrogen-bond geometry (Å, º)

**Table:**

*D*—H···*A*	*D*—H	H···*A*	*D*···*A*	*D*—H···*A*
C34—H34···Cl1^i^	0.93	2.99	3.631 (5)	127
C35—H35···Cl1^i^	0.93	2.97	3.616 (4)	128

Symmetry code: (i) *x*, *y*−1, *z*.
